# The cerebellum is involved in implicit motor sequence learning

**DOI:** 10.3389/fnins.2024.1433867

**Published:** 2024-12-06

**Authors:** Mahyar Firouzi, Kris Baetens, Catalina Duta, Chris Baeken, Frank Van Overwalle, Eva Swinnen, Natacha Deroost

**Affiliations:** ^1^Brain, Body and Cognition Research Group, Faculty of Psychology and Educational Sciences, Vrije Universiteit Brussel, Elsene, Belgium; ^2^Rehabilitation Research Group, Department of Physiotherapy, Human Physiology and Anatomy, Vrije Universiteit Brussel, Jette, Belgium; ^3^Center for Neurosciences (C4N), Vrije Universiteit Brussel, Elsene, Belgium; ^4^Brussels Human Robotic Research Center (BruBotics), Vrije Universiteit Brussel, Jette, Belgium; ^5^Department of Psychiatry and Medical Psychology, Ghent University, University Hospital Ghent (UZ Ghent), Ghent, Belgium; ^6^Department of Psychiatry, Faculty of Medicine and Pharmacy, Vrije Universiteit Brussel (VUB), University Hospital Brussel (UZ Brussel), Jette, Belgium

**Keywords:** cerebellum, implicit motor sequence learning, non-invasive brain stimulation, tDCS, motor learning, basal ganglia

## Abstract

**Background:**

Implicit motor sequence learning (IMSL) is a cognitive function that allows us to execute multiple movements in a specific sequential order and plays a crucial role in our daily functional activities. Although the role of the basal ganglia network in IMSL is well-established, the exact involvement of the cerebellar network is less clear.

**Aim:**

Here, we aimed to address this issue by investigating the effects of cerebellar transcranial direct-current stimulation (tDCS) on IMSL.

**Methods:**

In this sham-controlled, crossover study in 45 healthy young adults, we used mixed-effects models to analyze sequence-specific (primary outcome) and general learning effects (secondary outcome) in the acquisition (during tDCS), short- (five minutes post-tDCS) and long-term consolidation (one week post-tDCS) phases of IMSL, as measured by the serial reaction time (SRT) task.

**Results:**

Analyses based on response times (RTs) revealed that anodal tDCS over the cerebellum significantly increased sequence-specific learning during acquisition, compared to sham (anodal: *M* = 38.24 ms, sham: *M* = 26.78 ms, *p* = 0.032); did not affect general learning; and significantly slowed overall RTs (anodal: *M* = 362.03 ms, sham: *M* = 356.37 ms, *p* = 0.049). Accuracy-based analyses revealed that anodal tDCS reduced the probability of correct responses occurring in random trials versus sequential trials by 1.17%, *p* = 0.009, whereas sham tDCS had no effect, *p* = 0.999.

**Conclusion:**

Our finding of enhanced sequence-specific learning, but not general learning, suggests that the cerebellar network not only plays a role in error correction processes, but also serves a sequence-specific function within the integrated motor learning network that connects the basal ganglia and cerebellum.

## 1 Introduction

Motor sequence learning is defined as the inherent ability to learn sequential actions (Keele et al., 2003). It is a hallmark function of motor skill that enables humans to execute multiple movements in a specific sequential order and plays a key role in daily functional activities such as reaching, dressing, walking, driving and playing musical instruments (Firouzi et al., 2021a). To an important degree, serial motor behavior can be acquired implicitly, which means that learning of the sequential order is incidental and not dependent on awareness (Kantak et al., 2012; Robertson, 2007). For example, driving a car involves a chain of actions performed in a specific order, initially requiring explicit awareness and attention. Over time, with practice, these actions become automatized, and the act of driving requires little explicit awareness. The sequential nature of these actions becomes challenging to verbalize, illustrating that a part of learning occurs implicitly (Cleeremans et al., 1998).

This so-called implicit motor sequence learning (IMSL) is classically investigated by means of the serial reaction time (SRT) task, which involves learning of a sequence of key presses in response to a sequence of corresponding visual targets (e.g., 132,342,134,142, with 1–4 referring to the leftmost, left, right and rightmost target locations) (Hashemirad et al., 2016; Savic and Meier, 2016). With training, overall response times (RTs) decrease, representing a general learning effect (secondary outcome) that encompasses both the non-sequence-specific and sequence-specific learning component of IMSL. However, inconspicuous interruptions of the sequence (e.g., in a random block) lead to a relative increase in RTs compared to preceding and subsequent sequential blocks, denoting the sequence-specific learning effect (primary outcome) (Firouzi et al., 2023). Whereas general learning effects encompass effects that do not directly reflect sequence learning, such as general motor facilitation effects, exploring sequence-specific learning effects allows us to assess how tDCS specifically affects the implicit acquisition and consolidation of the imposed sequence (Firouzi et al., 2023).

Learning of sequential abilities is thought to involve the striatum, cerebellum, supplementary motor, primary motor, premotor and dorsolateral prefrontal cortices (Meissner et al., 2016). This process is supposedly mediated by two sets of brain circuits, working in parallel: the cortico-basal ganglia-thalamo-cortical circuit (basal ganglia network) and the cerebello-thalamo-cortical circuit (cerebellar network) (Doyon et al., 2003; Hikosaka et al., 2002). The specialized role of the basal ganglia network in IMSL has been well-established by numerous brain imaging studies (Doyon et al., 2009; Hikosaka et al., 2002; Rauch et al., 1997). Crucially, and in line with the notion that IMSL relies heavily on this network, this type of learning is impaired in individuals with basal ganglia dysfunction (Wilkinson et al., 2009). This includes patients with focal basal ganglia lesions (Vakil et al., 2000), Huntington’s disease (Kim et al., 2004; Knopman and Nissen, 1991; Willingham and Koroshetz, 1993) and Parkinson’ disease (Deroost et al., 2006, 2018; Firouzi et al., 2021b; Kelly et al., 2004; Wilkinson et al., 2009). In contrast to the basal ganglia network, however, the cerebellar network’s involvement is less clear (Baetens et al., 2020; Janacsek et al., 2020).

An influential model by Doyon and colleagues posits that the recruitment of these two networks is dependent on the stage of learning (Doyon et al., 2002; Penhune and Steele, 2012). In line with this model, neuroimaging studies attribute rapid performance improvements in the early learning or *acquisition* phase to both networks, but show a declining contribution of the cerebellum as task proficiency improves (Doyon et al., 2002; Floyer-Lea and Matthews, 2004, 2005; Penhune and Doyon, 2002, 2005). In contrast, the subsequent *consolidation* phase is suggested to be primarily mediated by striatal mechanisms. Finally, the basal ganglia network and parietal areas support the transition into the slow learning or *retention* phase, where performance becomes optimized, automated, and less attentionally demanding after extended practice (Doyon et al., 2002, 2009; Doyon and Benali, 2005; Penhune and Steele, 2012). Although this theoretical model describes the individual contributions of both networks to sequence learning, neuroanatomical studies suggest that the basal ganglia and cerebellum are interconnected during the acquisition of a novel motor sequence (Bostan et al., 2013; Bostan and Strick, 2018; Milardi et al., 2016), not only at the level of the cerebral cortex, but at the subcortical level as well, forming an integrated network (Bostan and Strick, 2018; Milardi et al., 2016).

While some studies suggest that the cerebellar network primarily serves a non-sequence-specific function, such as adjusting movement kinematics to sensory inputs (Penhune and Doyon, 2002, 2005), lesion studies with cerebellar patients show that the ability to sequence is the most adversely affected cognitive domain, pointing towards a sequence-specific function of the cerebellum (Tedesco et al., 2011). This is in line with the view that the cerebellum detects sequential patterns, generates internal models of sequential motor (and even non-motor) processes, and integrates action predictions with sensory feedback to fine-tune behaviour [cfr. *Sequence detection hypothesis*, Leggio and Molinari (2015) and Van Overwalle et al. (2019)].

Previous studies have employed non-invasive brain stimulation techniques, such as transcranial direct current stimulation (tDCS), to establish connections between specific brain structures and motor learning (Hashemirad et al., 2016; Kumari et al., 2019). tDCS involves applying a mild electrical current to the scalp, between minimally two electrodes (one anode and one cathode), which generally results in increased or decreased cortical excitability (Nitsche and Paulus, 2000; Polanía et al., 2012). Various tDCS studies have demonstrated the involvement of the primary motor cortex by influencing acquisition and consolidation of sequential knowledge in healthy adults (Cuypers et al., 2013; Firouzi et al., 2023; Kang and Paik, 2011; Kantak et al., 2012; Nitsche et al., 2003; Tecchio et al., 2010) and in patients with Parkinson’s disease (Firouzi et al., 2021a, 2021b). With regard to anodal cerebellar tDCS, positive effects are also reported rather consistently when looking at broader, non-sequence-specific measures of motor learning as measured by force field and visuomotor adaptation tasks [e.g., Galea et al. (2011), Hardwick and Celnik (2014), and Herzfeld et al. (2014); and review article by Kumari et al. (2019)]. However, more heterogeneous findings are reported across studies on the role of the cerebellum with regard to sequence-specific learning, with some SRT studies revealing positive effects on sequence-specific and general learning compared to sham (Ehsani et al., 2016; Ferrucci et al., 2013; Liebrand et al., 2020; Shimizu et al., 2017); but see (Jongkees et al., 2019; Ma et al., 2023) for null-findings. Importantly, however, some of these studies on cerebellar tDCS and sequence learning focused on either *explicit* aspects of motor sequence learning (Liebrand et al., 2020) or implicit aspects of *non-motor* sequence learning (Ma et al., 2023). Others only considered non-sequence specific measures of motor learning (Ehsani et al., 2016; Samaei et al., 2017), or solely assessed effects on acquisition and short-term consolidation (Ferrucci et al., 2013).

In sum, there is considerable debate surrounding the role of the cerebellum in IMSL, particularly regarding its sequence-specific involvement. Additionally, there are lingering questions regarding the extent of its contribution beyond the initial acquisition phase, notably in terms of long-term consolidation of sequential knowledge. Given the controversy in the literature, this study aimed to clarify the sequence-specific role of the cerebellum in the acquisition (during tDCS), short-term (five minutes post-tDCS) and long-term consolidation (one week post-tDCS) phases of IMSL, as measured by the SRT task.

We hypothesized that, in line with previous studies (Ferrucci et al., 2013; Liebrand et al., 2020; Shimizu et al., 2017), cerebellar tDCS would enhance acquisition, but not consolidation of sequence-specific and general learning effects. This finding would corroborate the notion that the cerebellum is primarily involved in the earliest stages of IMSL (Doyon et al., 2002; Floyer-Lea and Matthews, 2004, 2005; Penhune and Doyon, 2002, 2005). Given the established role of the cerebellum in error correction processes, particularly during early learning (Ito, 2000; Steele and Penhune, 2010), we also hypothesized that anodal tDCS would improve accuracy, particularly during the acquisition phase compared to sham.

## 2 Methods

### 2.1 Study design

In this single-blind, sham-controlled, counterbalanced study, all participants received both anodal and sham tDCS in a random order (concealed for the participants). Ethical approval was obtained from the Medical Ethics Committee of the University Hospital Brussels (identifier: B1432020000011).

### 2.2 Participant recruitment and inclusion criteria

Forty-five healthy young adults, aged 18 to 35 years, were recruited through social media and flyers. Potential candidates were excluded if they presented with a history of neurological and/or recent musculoskeletal diseases that could hamper the execution of the SRT task or known contra-indications for tDCS (deep brain stimulator; pacemaker; head wound; skin condition of the scalp; a history of epilepsy).

### 2.3 Transcranial direct-current stimulation

Soterix Medical’s 1×1 Low Intensity Direct Current Stimulator (Soterix Medical Inc., New York, USA) was used to apply tDCS to the cerebellum. Direct-current was delivered through a pair of identical square rubber electrodes placed in rectangular saline-soaked sponges (size 35 cm^2^). For both stimulation conditions (anodal/sham), the anode was centered on the median line 2 cm below the inion and the cathode was placed on the right upper arm, over the deltoid muscle (Ferrucci et al., 2012, 2013). The current was gradually increased from 0 mA to 2 mA in 60 s, but only maintained for the duration of the SRT task (median duration + − 17 min) in the anodal condition. In the sham condition the current was gradually decreased to 0 mA again after the initial ramp-up. This ramping-up and -down was repeated at the end of the stimulation session to optimize participant blinding.

### 2.4 Serial reaction time task

**Figure 1:**
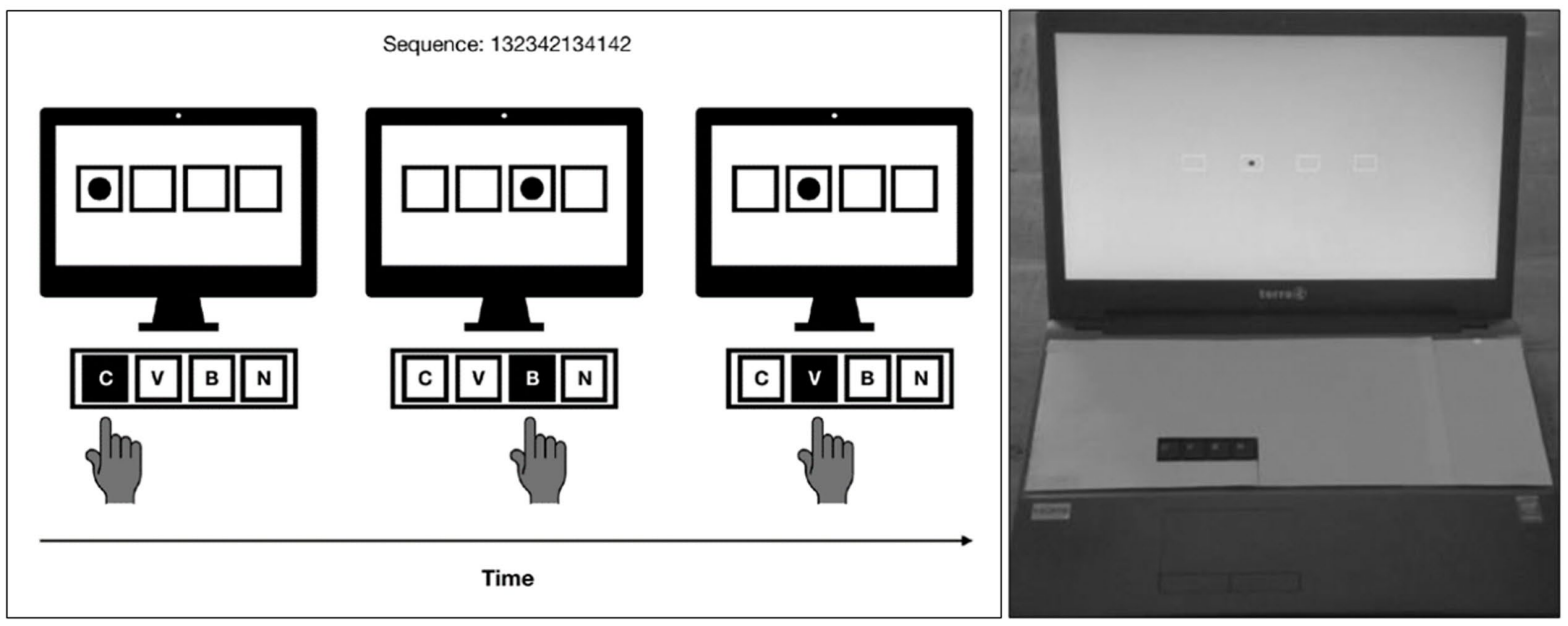
Schematic representation of the SRT task. [Left] On each trial, participants had to react as quickly as possible to the target location by pressing the spatially corresponding response key with the index finger of their dominant hand - in this example of three consecutive trials: C, B, V. Unknown to the participants, the target location followed a repeating sequence (e.g., 132342134142, with 1–4 referring to the target locations from left to right). [Right] Only the response keys C, V, B, N were visible to the participants. Taken from Firouzi et al. (2024).

The SRT task, identical to (Deroost et al., 2018; Firouzi et al., 2023, 2024; Firouzi, Van Herk, et al., 2021), was performed on laptops using E-Prime® software (Psychology Software Tools, Inc., Pittsburgh, PA, USA), see Figure 1. Participants were instructed to press the response keys C, V, B and N for, respectively, a leftmost, left, right and rightmost target, using the index finger of their dominant hand. Only the response keys were visible to the participants, all other keys were covered. Correct responses were followed by the next stimulus, which appeared after a response–stimulus interval (RSI) of 300 ms. In case of erroneous responses, an error message appeared for 1,050 ms, followed by the next stimulus.

For the acquisition (concurrent with tDCS) and long-term consolidation (one week post tDCS) phases, the full-version SRT task consisted of eight blocks of 72 trials (sequential Blocks 1–6 and 8, random Block 7), preceded by a random practice block consisting of 72 trials. There was an optional thirty-second break between consecutive blocks.

Unknown to the participants, the order of the target (i.e., black dot) locations followed a repeating sequence in Blocks 1 through 6 and Block 8 (e.g., 132,342,134,142, with 1–4 referring to the target locations from left to right). The rationale is that RTs decrease with repetition of the sequence, denoting a general learning effect (secondary outcome measure of IMSL), which reflects more general aspects of motor skill acquisition. However, the relative increase in RTs when the sequence is interrupted in random Block 7 relative to preceding and subsequent sequential blocks, denotes a sequence-specific learning effect (primary outcome measure of IMSL).

**Figure 2:**
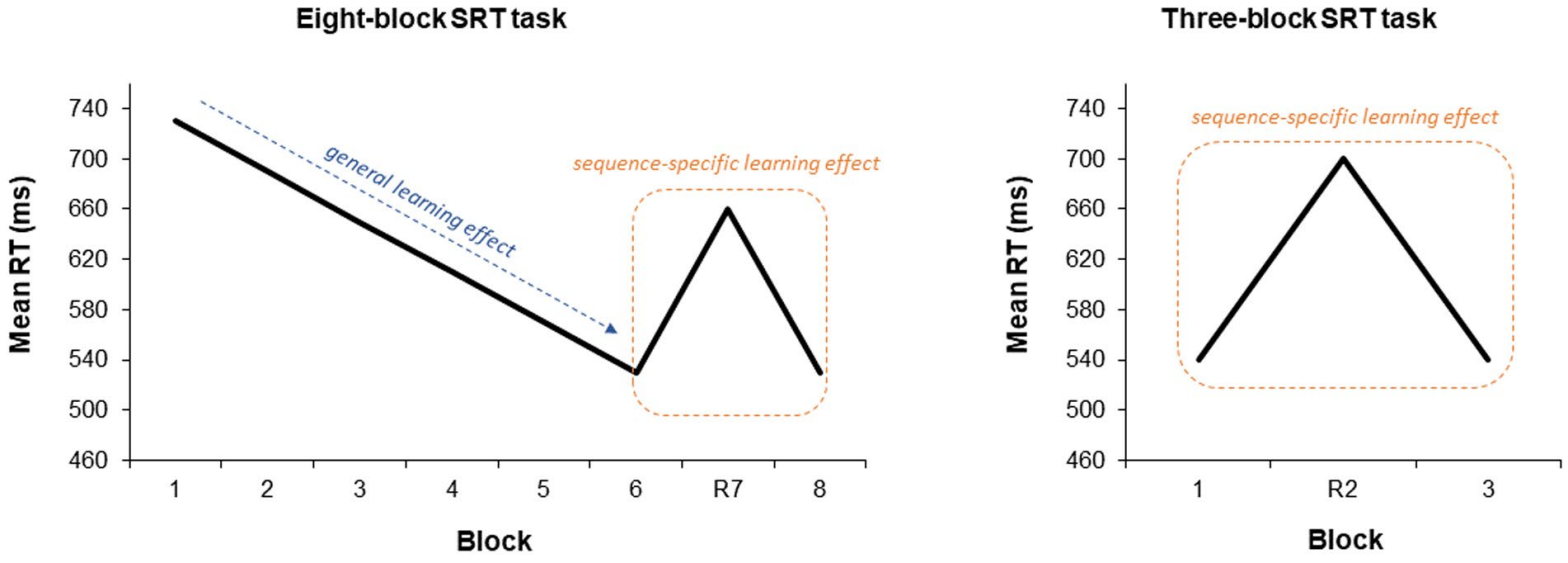
Visualization of sequence-specific and general learning effects in the SRT task. Visualization of SSLE (primary outcome) and GLE (secondary outcome) in the eight- (used during acquisition and long-term consolidation) and three-block (used during short-term consolidation) SRT tasks. The decrease in RTs with repetition of the sequence across sequential blocks reflects general learning. The relative increase in RTs when the sequence is interrupted in the random block relative to preceding and subsequent sequential blocks reflects SSLE. The three-block task does not allow for analysis of GLE, as the second random block introduces noise in the data. Taken from Firouzi et al. (2024).

Short-term consolidation (five minutes post tDCS) was assessed by means of a shortened version of the task. This version consisted of only three blocks (sequential blocks 1 and 3, random block 2), since it was administered shortly after the full-length task and does not allow for analysis of general learning effects, as the second random block introduces noise in the data. See Figure 2 for a representation of these outcomes in the eight- and three-block SRT tasks.

### 2.5 Experimental procedure

**Figure 3:**
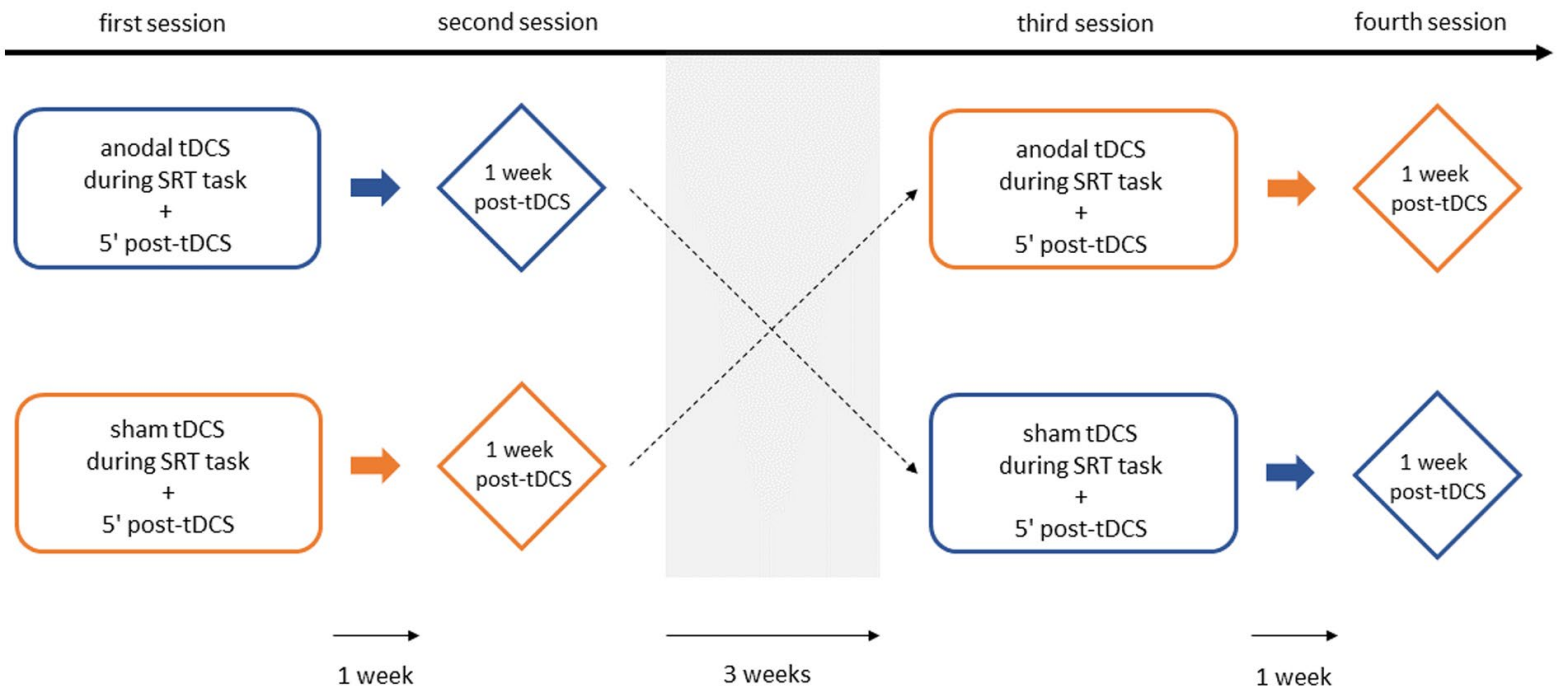
Schematic representation of the experimental design. Representation of the sham-controlled, counter-balanced experimental design.

Figure 3 schematically represents the experimental procedure. Following a screening session, in which baseline demographic characteristics (sex, age, dominant hand, education level) were collected, all participants were seen four times over the course of five weeks. During the first and third sessions, the full-version SRT task was performed with concurrent anodal or sham tDCS (*acquisition phase*). Five minutes post tDCS, the shortened SRT task was carried out without application of tDCS (*short-term consolidation phase*). The second and fourth sessions were scheduled one week after the first and third sessions, respectively. These sessions involved the same full-version SRT task conducted one week earlier, but without the application of tDCS (*long-term consolidation phase*). To control for carry-over effects between the two tDCS conditions, a washout period of at least three weeks was incorporated (in line with Boggio et al., 2007; Thair et al., 2017; Vaseghi et al., 2015), after which the experimental procedure was repeated with the opposite stimulation condition. Additionally, the stimulation condition order was counterbalanced across participants to help mitigate potential bias from such effects. Since explicit awareness of the sequence can interfere with implicit learning (Destrebecqz et al., 2005; Fletcher et al., 2005), participants filled out a post-SRT task questionnaire after the final session to assess whether they had become aware of the sequential nature of the task.

### 2.6 Outcome measures and statistical analysis

All statistical analyses were carried out using R (version 4.2.1) and IBM SPSS Statistics (version 28). The *lme4* (version 1.1–30) and *car* packages for R were used to fit generalized linear mixed-effect models (GLMMs) and to conduct type III Wald tests for the fitted GLMMs, respectively (Fox and Weisberg, 2011; Jongkees et al., 2019). The *emmeans* package was used to perform post-hoc tests for significant interaction effects (De Rosario-Martinez, 2015; Jongkees et al., 2019), and the *ggplot2* package was used for the graphical representation of those effects (Jongkees et al., 2019; Wickham, 2016). Level of significance was set at *α* = 0.05. Contrasts were Tukey-adjusted.

The GLMM approach was chosen as it allows us to adequately account for the structure in the data, does not require data averaging, nor discarding of data in case of subject attrition, in contrast to traditional ANOVA methods (Lo and Andrews, 2015; Thalmann et al., 2017).

#### 2.6.1 Baseline demographical variables

Pearson correlation analyses, Bonferroni-corrected for multiple comparisons, were performed to investigate if IMSL (i.e., difference between RT random block and mean RT of adjacent sequential blocks) correlated with demographical variables (sex, age, dominant hand, education level). If assumptions for parametrical testing were violated, the non-parametric alternative Spearman’s Rho was calculated.

#### 2.6.2 Response times

Analyses of the SRT task performance were based on response times (RTs). Practice trials (16.89% of all data), RTs shorter than 100 ms (1.14%), RTs longer than 2000 ms (0.09%), and erroneous responses (2.86%) were excluded from the analyses.

Sequence-specific learning effects (primary outcome) were defined as significant differences in RTs between random and sequential blocks, and were analyzed by fitting a series of GLMMs with inverse Gaussian family and log link to the RTs of each trial (Bates et al., 2015; Jongkees et al., 2019; Lo and Andrews, 2015). Only random blocks and their adjacent sequential blocks (i.e., all blocks for the shortened three-block SRT task and blocks 6, 7, and 8 for the full-version eight-block tasks) were included in these analyses to circumvent convergence issues and unworkable complexity in the model. The fixed factors *Stimulation condition* (anodal, sham), *Session* (during tDCS, five minutes post tDCS, one week post tDCS) and *Block type* (sequential, random) were included as predictors, with interactions among them. To account for between-subject variance in RTs, random intercepts for participants were added, and a maximal random effects structure was used for the slopes of the predictive variables (Barr et al., 2013).

General learning effects (secondary outcome) were analyzed by fitting a series of GLMMs with inverse Gaussian family and log link to the RTs of each trial in the sequential blocks (i.e., Blocks 1, 2, 3, 4, 5, 6, 8) of the full-version SRT tasks. General learning effects were not analyzed for the shortened SRT task as it consisted of three blocks only. *Stimulation condition* (anodal, sham), *Session* (during tDCS, one-week post tDCS) and the second degree (i.e., quadratic and linear) polynomials of *Block* (1, 2, 3, 4, 5, 6, 8) were included as fixed factors. Interactions between these factors and random intercepts for *Participants* were again added to the models.

For both outcomes, the goodness of fit was assessed by means of Akaike Information Criteria (AIC).

#### 2.6.3 Accuracy

As error percentages in the SRT task are generally small and likely to reflect motor rather than predictive errors (Urry et al., 2015), accuracy is a less sensitive measure of IMSL. However, given the well-established role of the cerebellum in error correction and to ensure comprehensive analysis, we also fitted a series of GLMMs to the accuracy of each trial. Given the binary nature of the dependent variable (false/correct), we employed binomial logistic regressions. In all other respects, these GLMM analyses were conducted analogously to the RT analyses.

#### 2.6.4 Sequence awareness

Following the final session, participants completed a post-SRT task questionnaire to assess explicit awareness of the sequential nature of the task. First, participants were asked if they noticed a repetitive pattern in the task or believed that the stimuli appeared randomly. If participants indicated subjective sequence awareness, they were subsequently prompted to freely recall and replicate a maximum number of sequence elements as accurately as possible. To obtain a more objective assessment of explicit sequential awareness, participants were given a reproduction score on a scale from zero to twelve. This score was determined by the maximum number of sequence elements they successfully reproduced in the correct order.

## 3 Results

### 3.1 Participants

Out of 45 participants, 42 (93.33%) completed all sessions. Data was partially incomplete for three of these 42 participants due to technical errors. No data was discarded due to subject attrition or missing data as this is not required when using mixed-effects models (Lo and Andrews, 2015). Baseline demographical characteristics and sequential awareness/reproduction scores for all 45 participants are summarized in Table 1.

**Table 1:** Baseline demographical variables and sequential reproduction score.

Variable	Frequencies/Mean ± SD
Sex (male:female)	22:23
Age (years)	24.31 (± 3.94)
Level of education* (1:2:3:4)	18:9:12:6
Dominant hand (L:R)	7:38
Subjective sequential awareness (Y/N)	31:14
Reproduction score	3.94 (± 1.18)

L, left; R, right; Y, yes; N, no.

*Level of education: based on an ordinal four-point scale: (1) lower secondary education; (2) professional (college) bachelor’s degree; (3) academic (university) bachelor’s degree; (4) academic (university) master’s degree.

### 3.2 Response times

#### 3.2.1 Sequence-specific learning effects

The best fitting GLMM for the analysis of sequence-specific learning effects included interactions between the predictors *Stimulation condition* (anodal, sham), *Session* (during tDCS, five minutes post tDCS, one week post tDCS), and *Block type* (*sequential*, *random*) as fixed factors, and an intercept and slopes for *Stimulation condition*, *Session*, and their interaction within *Participants* as the maximally supported random effects structure.

**Figure 4:**
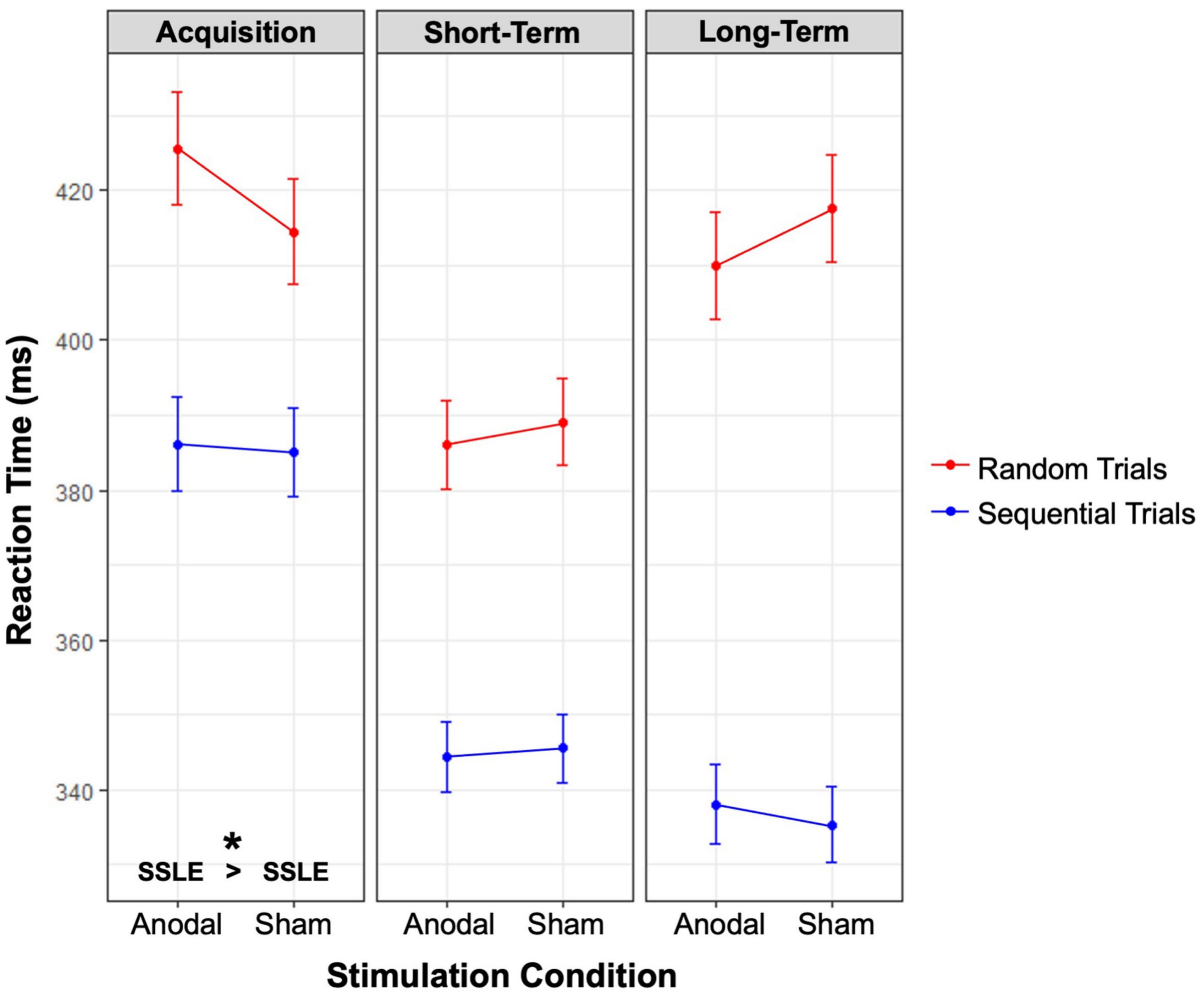
Sequence-specific learning effects. Interaction effects between the factors stimulation condition, block type, and session. Estimated marginal means are displayed. Red lines denote random trials, blue lines denote sequential trials. Sequence-specific learning occurred at each point in time, and for each condition. SSLE was significantly larger in the anodal condition compared to sham during acquisition, as marked by an asterisk at the bottom of the leftmost panel. Abbreviations: SSLE sequence-specific learning effects.

The three-way interaction between *Stimulation condition, Session,* and *Block type* was significant [*X*^2^ (2, *N* = 45) = 6.10, *p* = 0.047], see Figure 4. Although sequence-specific learning effects occurred in both stimulation conditions and in each learning phase, contrasts revealed that anodal tDCS resulted in a significantly larger effect during acquisition (*M* = 38.24 ms, *SE* = 7.44), compared to sham (*M* = 26.78 ms, *SE* = 6.53); *p* = 0.032, *z* = 2.15, *ratio* = 1.03, *SE* = 0.01. At a qualitative level, this significantly larger effect (i.e., the difference between RTs in random versus sequential trials) appears to be attributable to slowed responses in random trials, rather than faster responses in sequential trials; however, neither of these contrasts were significant, *p* = 0.469, *z* = 1.04 and *p* = 0.999, *z* = 1.01, respectively. Sequence-specific learning effects did not differ significantly between stimulation conditions at short-term (*p* = 0.463, *z* = 0.73) and long-term consolidation (*p* = 0.183, *z* = −1.33). Although learning increased with each session, regardless of *Stimulation condition* [*X*^2^ (2, *N* = 45) = 63.95, *p* < 0.001], follow-up contrasts of the three-way interaction revealed that the increase from acquisition to long-term consolidation was significantly smaller in the anodal condition (*MD* = 31.48 ms, *SE* = 9.77), compared to sham (*MD* = 48.85 ms, *SE* = 8.93); *p* = 0.014, *z* = 2.46, *ratio* = 1.04, *SE* = 0.02.

To summarize, anodal cerebellar tDCS induced larger sequence-specific learning effects during acquisition, compared to sham. Although follow-up contrasts do not provide conclusive evidence, this effect seems to be driven by slowed responses in random trials. Additionally, anodal tDCS led to a smaller increase in sequence-specific learning effects from acquisition to long-term consolidation, seemingly hindering the consolidation of this knowledge over time. However, this finding can be more plausibly explained by ceiling effects. This is supported by the finding of similar magnitudes of learning effects between stimulation conditions at long-term consolidation.

#### 3.2.2 General learning effects

The best fitting GLMM for the analysis of general learning effects included interactions between the predictors *Stimulation condition* (anodal, sham)*, Session* (during tDCS, five minutes post tDCS, one week post tDCS), and quadratic polynomials of *Block* (1, 2, 3, 4, 5, 6, 8) as fixed factors, and an intercept and slopes for *Stimulation condition, Session,* and their interaction within *Participants* as the maximally supported random effects structure.

**Figure 5:**
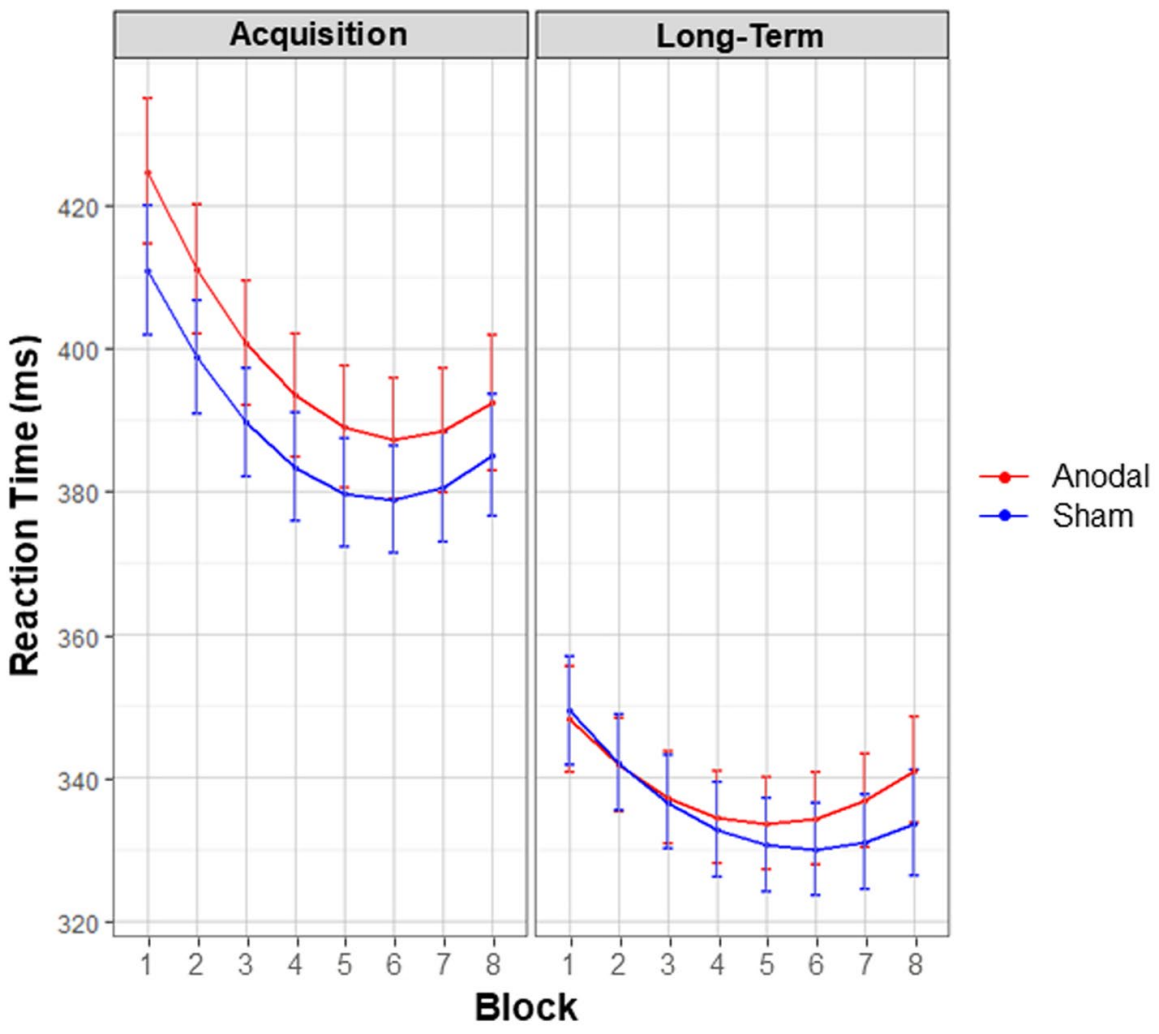
General learning effects. Interaction effects between the factors stimulation condition, block, and session. Estimated marginal means are displayed. Red lines denote anodal tDCS, blue lines denote sham tDCS. Although anodal tDCS slowed overall motor performance, regardless of other factors, there was no significant influence of stimulation condition on general learning (secondary outcome).

The three-way interaction between *Stimulation condition*, *Session* and polynomials of *Block* showed a trend towards significance [*X*^2^ (2, *N* = 45) = 5.79, *p* = 0.055], see Figure 5. An exploratory look at the contrasts revealed no significant differences in general learning effects between conditions during acquisition (*p* = 0.648, *z* = −1.17) or long-term consolidation (*p* = 0.156, *z* = 2.09). The significant two-way interaction between *Session* and polynomials of *Block* [*X*^2^ (2, *N* = 45) = 27.01, *p* < 0.001] revealed that, regardless of condition, smaller general learning effects occurred at long-term consolidation compared to acquisition. However, these smaller effects can be attributed to overall faster RTs at long-term consolidation, as demonstrated by the significant main effect of *Session* [*X*^2^ (1, *N* = 45) = 278.30, *p* < 0.001]. Finally, the main effect of *Stimulation condition* [*X*^2^ (1, *N* = 45) = 3.87, *p* = 0.049] showed that overall RTs were marginally significantly slower in the anodal condition (*M* = 362.03 ms, *SE* = 3.30), compared to sham (*M* = 356.37 ms, *SE* = 2.97).

In sum, anodal cerebellar tDCS marginally significantly slowed overall motor performance (i.e., overall higher RTs, regardless of blocks), and did not affect general learning (i.e., progressive decrease in RTs, across sequential blocks).

### 3.3 Accuracy

Regardless of all other factors, participants responded with 97.13% accuracy (SD = 0.17%) in the anodal condition and 97.22% (SD = 0.17%) in the sham condition.

#### 3.3.1 Sequence-specific learning effects

The best fitting GLMM for the analysis of sequence-specific learning effects based on accuracy data included interactions between the predictors *Stimulation condition* (anodal, sham)*, Session* (during tDCS, five minutes post tDCS, one week post tDCS), and *Block type (sequential, random)* as fixed factors, and an intercept and slopes for *Stimulation condition* and its interaction within *Participants* as random effects structure.

The significant main effect of *Block type* [*X*^2^ (1, *N* = 45) = 14.20, *p* < 0.001] indicated that, as to be expected, participants had a higher estimated probability of giving a correct response in sequential trials [probability = 97.95%; 95% CI = (97.51, 98.31%)] compared to random trials [probability = 97.08%; 95% CI = (96.46, 97.60%)].

**Figure 6:**
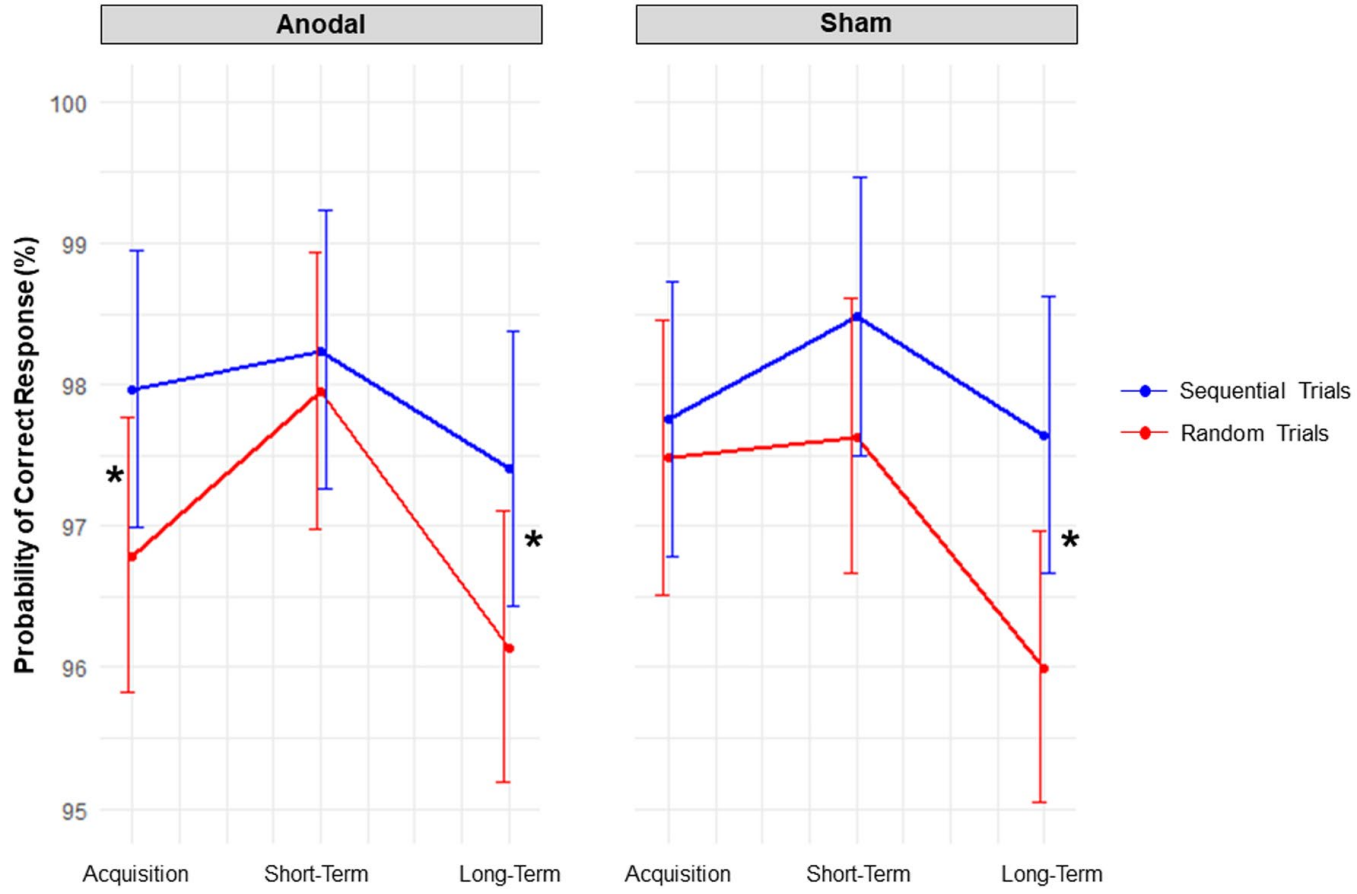
Accuracy. Interaction effects between the factors stimulation condition, block, and session. Red lines denote random trials, blue lines denote sequential trials. Probabilities of correct responses are displayed. Error bars represent 95% confidence intervals. Asterisks indicate significant contrasts.

The three-way interaction between *Stimulation condition*, *Session*, and *Block type* [*X*^2^ (2, *N* = 45) = 6.79, *p* = 0.034] was significant. Contrasts revealed that, during acquisition, anodal tDCS decreased the probability of correct responses occurring in random trials versus sequential trials by 1.17%; *p* = 0.009, *z* = −3.77, *odds ratio* = 0.63, *SE* = 0.08; whereas sham tDCS had no effect; *p* = 0.999, *z* = −0.91, *odds ratio* = 0.89, *SE* = 0.12; see Figure 6. However, when comparing the probabilities of correct responses in random trials between anodal and sham tDCS, no significant difference was found; *p* = 0.918, *z* = −1.58, *odds ratio* = 0.78, *SE* = 0.12. At short-term consolidation, there was no impact of either stimulation condition, both *p*’s > 0.050. At long-term consolidation, the probability of correct responses occurring in sequential versus random trials was 1.26% higher for anodal tDCS, *p* = 0.020, *z* = −3.55, *odds ratio* = 0.66, *SE* = 0.08; and 1.66% higher for sham tDCS; *p* < 0.001, *z* = −4.67, *odds ratio* = 0.58, *SE* = 0.07. However, when comparing these effects between stimulation conditions, no significant difference was found, *p* = 0.397, *z* = 0.85, *odds ratio* = 1.15, *SE* = 0.19.

In sum, anodal tDCS potentially exerted a negative influence on accuracy in random trials compared to sequential trials, whereas sham tDCS did not.

#### 3.3.2 General learning effects

The best fitting GLMM for the analysis of general learning effects based on accuracy data included interactions between the predictors *Stimulation condition* (anodal, sham)*, Session* (during tDCS, five minutes post tDCS, one week post tDCS), and quadratic polynomials of *Block* (1, 2, 3, 4, 5, 6, 8) as fixed factors, and an intercept and slopes for *Stimulation condition, Session,* and their interaction within *Participants* as the maximally supported random effects structure. This analysis did not reveal any significant main or interaction effects.

### 3.4 Sequence awareness

Thirty-one out of 45 participants (68.89%) subjectively reported that they believed the stimulus did not appear randomly on the screen (i.e., subjective sequence awareness). However, when asked to freely recall and reproduce a maximum number of sequence elements as accurately as possible, 16 (35.56%) participants made the attempt. These 16 participants were able to reproduce an average of 4.20 (*SD* = 1.68) out of 12 elements of the sequence in the correct order.

## 4 Discussion

In this study, we aimed to clarify the role of the cerebellum in the acquisition (during tDCS), short-term (five minutes post-tDCS) and long-term (one week post-tDCS) consolidation phases of IMSL, as measured by the SRT task. In what follows, the main findings are summarized first, and then discussed in more detail.

The impact of tDCS on IMSL was found to be dependent on the specific learning type and the stage at which it occurred. As hypothesized, anodal tDCS significantly increased sequence-specific learning effects (primary outcome), solely during the acquisition phase. However, it should be noted that the interaction effects of interest were of modest significance (i.e., just below the 0.05 threshold). This observation is in line with previous research [Ferrucci et al., 2013; Shimizu et al., 2017, but see Jongkees et al. (2019) and Ma et al. (2023) for null-findings]. As expected, no stimulation effects were found at short- and long-term consolidation. In terms of general learning effects (secondary outcome), which involve the progressive reduction in response times across sequential blocks, we observed no discernible influence from anodal tDCS, refuting our initial hypothesis. Intriguingly, anodal tDCS impacted accuracy in random trials during acquisition and slightly slowed down overall motor performance during the SRT task. While the latter observation contrasts with previous reports of faster responses following a similar cerebellar tDCS protocol (Ma et al., 2023), these divergent findings are likely due to group differences, with Ma et al. (2023) employing a between-subjects design versus our within-subjects approach. Alternatively, this overall slowed performance might reflect a modulatory effect of anodal cerebellar tDCS on cerebellar-brain inhibition (CBI). Potentially, anodal stimulation increased CBI, which in turn inhibited motor cortex excitability [in line with, e.g., Katagiri et al. (2021)].

Our finding of enhanced acquisition of sequence-specific learning effects under anodal cerebellar tDCS is in line with theoretical models asserting that the cerebellum (a) serves a sequence-specific function (Leggio and Molinari, 2015; Tedesco et al., 2011; Van Overwalle et al., 2019), and (b) primarily facilitates the early acquisition of sequential knowledge (Doyon et al., 2002; Floyer-Lea and Matthews, 2004, 2005; Penhune and Doyon, 2002, 2005). Crucially, the presence of enhanced sequence-specific, but not general learning effects, starkly contrasts with earlier claims that argued against the involvement of the cerebellum in sequence learning itself (Janacsek et al., 2020). Although, qualitatively, the observed positive impact of anodal cerebellar tDCS on acquisition of sequence-specific learning effects appears to be driven by slowed responses in random trials (see Figure 4), our analyses do not offer conclusive evidence regarding this contrast. Regardless, the observed positive effect clearly cannot be attributed to faster responses in sequential trials (see Figure 4).

Within the context of IMSL, the distinction between sequence-specific learning effects driven by slowed RTs in random trials versus faster RTs in sequential trials is not trivial. For instance, while faster responses in sequential trials would unambiguously indicate more efficient acquisition and execution of the learned sequence, this pattern was not observed in the present study. Instead, the presence of slowed responses exclusively in random trials may indicate that anodal cerebellar tDCS increases cognitive rigidity or inflexibility in adapting to unexpected stimuli, without necessarily impacting the level of sequential knowledge. On the other hand, we posit that slowed RTs in random trials do not negate the possibility of a positive impact on sequential learning processes. Rather, these delayed responses resulting from sequence disruption may be indicative of participants reacting to the sudden disruption of the learned sequence, and hence, reflect enhanced learning processes. Interestingly, we observed a very similar pattern of results in two previous tDCS-IMSL studies, in which application of anodal tDCS over the primary motor cortex analogously induced larger sequence-specific learning effects in healthy young adults (Firouzi et al., 2023), and in individuals with Parkinson’s disease with mild cognitive impairments (Firouzi et al., 2024). Although the qualitatively negative effect on RTs in random trials was insignificant in the present and both our previous studies, the consistency of this finding across both stimulation sites (i.e., cerebellum and primary motor cortex) and populations (i.e., healthy young adults and Parkinson’s disease) is remarkable.

Regardless of the potential explanations for the slowed responses in random trials mentioned above, the statistical findings reported here and in our previous studies support the conclusion that anodal tDCS can enhance sequence-specific learning effects during acquisition compared to sham.

Besides the observed effects on RT-based measures, it is worth noting that anodal cerebellar tDCS significantly impaired accuracy in random trials during acquisition, whereas our previous studies on tDCS of the motor cortices left accuracy unaffected. Although our initial hypothesis presumed a positive effect of anodal tDCS on accuracy, we did not make an *a priori* distinction between potential effects in random versus sequential trials. Analogous to our RT-based results, the occurrence of such effects *exclusively* in random trials could imply that anodal cerebellar tDCS hinders flexible responses to unexpected stimuli and increases cognitive rigidity. Alternatively, it may indicate that participants react more strongly to sequence disruption, potentially reflecting enhanced learning processes. Irrespective of its polarity, this effect on accuracy corroborates the established role of the cerebellum in error correction processes, particularly in the earliest stages of learning (Ito, 2000; Steele and Penhune, 2010). Further research is warranted to explore whether this seemingly negative impact on accuracy in random trials can be attributed to less flexible responses to unexpected stimuli during anodal cerebellar tDCS. For instance, a simultaneous tDCS-EEG study could reveal whether the P1 event-related potential (ERP) component, which is known to decrease following predictable events and increase following random events, is significantly impacted by cerebellar tDCS (Firouzi et al., 2023; Lum et al., 2019; Verleger and Śmigasiewicz, 2016). The observation of further decreases in P1 following sequential trials would corroborate the involvement of the cerebellum in IMSL. Conversely, the observation of further increases in P1 following random trials would support the hypothesis that anodal cerebellar tDCS increases cognitive rigidity.

Prior studies investigating the effects of cerebellar tDCS on implicit SRT tasks have yielded divergent outcomes, likely due to heterogeneous tDCS protocols and inconsistent operationalization of IMSL outcomes. For instance, our findings align with those of Ferrucci et al. (2013), who observed a positive effect of an identical cerebellar tDCS protocol on the implicit acquisition of sequence-specific knowledge. In contrast, Shimizu et al. (2017) utilized the same stimulation protocol, but with cathodal placement on the cheek instead of the upper arm, and reported no effect on the same outcome. Voegtle et al. (2023) directly compared effects of right cerebellar and left primary motor cortex tDCS on the SRT task and reported slowed responses, only during cerebellar tDCS. Although this seems to align with our finding of slowed overall motor performance during cerebellar tDCS, the authors attribute their observation to a specific suppression of IMSL (i.e., slower RTs in sequential, but not random trials), whereas our results indicate enhanced IMSL. Finally, one motor SRT study (Jongkees et al., 2019) and one social/cognitive SRT study (Ma et al., 2023) did not identify any effects of anodal cerebellar tDCS on sequence-specific learning. Interestingly, Jongkees et al. (2019) even tentatively suggest a detrimental impact on consolidation due to the inhibitory influence exerted by the cerebellum on the motor cortices [cerebellar-brain inhibition; see, e.g., Fernandez et al. (2018)].

Based on the findings summarized here, the cerebellar tDCS protocol employed by Ferrucci et al. (2013) and in the present study appears to induce rather consistent positive effects on IMSL. We therefore recommend using the same montage in future research on cerebellar tDCS and IMSL.

Taken together, the behavioral observations described here and in earlier work by, e.g., Ferrucci et al. (2013), Shimizu et al. (2017) and Liebrand et al. (2020), provide support for the framework proposed by Penhune and Steele (2012). This framework aligns distinct learning stages with the individual contributions of cortical and subcortical structures to IMSL. According to Penhune and Steele (2012), early acquisition heavily relies on the striatal system for forming predictive stimulus–response associations. Meanwhile, the cerebellum develops the optimal internal model for executing the sequence, and simultaneously engages in error correction and real-time movement control. The RT- and accuracy-based findings reported in the present study corroborate this dual role of the cerebellum in IMSL. While the primary motor cortex’s involvement spans all learning stages, with increasing engagement over extended practice, cerebellar activity decreases with learning. This is mirrored by the observation that, in this study, stimulation effects did not exceed the acquisition phase of IMSL. Notably, Penhune and Steele (2012) demonstrated that, although sequence-specific information becomes increasingly represented within the cortex, correlated activity between the primary motor cortex and the cerebellum also increases over time, suggesting that these regions form an integrated representation of the well-learned sequence (Penhune and Steele, 2012).

This complex interplay between the cerebellum and motor cortex is supported by the remarkable consistency of our findings across both our cerebellar and motor cortex tDCS montages (Firouzi et al., 2023, 2024). Consequently, we conclude that tDCS can beneficially modulate the integrated cortico-striato-cerebellar motor learning network that connects the basal ganglia and cerebellum at the cortical and subcortical level (Bostan et al., 2013; Bostan and Strick, 2018; Milardi et al., 2016). While cerebellar and motor cortex tDCS can alter cortical excitability directly, they can also indirectly modulate subcortical structures. Such subcortical effects of motor cortex tDCS on the basal ganglia network have been demonstrated before by Polanía et al. (2012), and presumably occur through pontine and thalamic synapses (Bostan et al., 2013), which ensure the closed-loop connection between the cerebellar and cerebral cortex.

Interestingly, a very significant proportion of participants in this study (31 out of 45; 69%) subjectively reported awareness of the sequential nature of the task. When asked to reproduce the sequence as accurately as possible (i.e., the more objective measure of explicit awareness), 16 out of 45 (36%) were able to reproduce an average of 4.2 out of 12 elements of the sequence in the correct order. In this regard, the potential impact of explicit sequential awareness on our findings cannot be ruled out. Furthermore, the interpretation of this potential impact is challenging, as both implicit and explicit sequence learning rely partially on the same neural networks (Wilkinson et al., 2009). Future studies should consider using a probabilistic sequence, where transitions between sequence elements are not strictly fixed but follow a likelihood pattern, potentially reducing the influence of explicit awareness on task performance.

A first limitation of this study was that we did not assess potential adverse effects of the stimulation. Future research should include such assessments to investigate potential differences in perceived side effects between anodal and sham stimulation conditions. Secondly, we did not evaluate the effectiveness of participant blinding to the stimulation condition. Moreover, experimenter blinding was not feasible with the tDCS device used in this study, which might have introduced unconscious bias risks. However, to mitigate this risk, experimenters remained out of view and refrained from interacting with participants during the task. Finally, since we did not combine tDCS with neuro-imaging techniques in the present study, we cannot draw conclusions regarding the specific impact of our cerebellar tDCS montage on the cerebello-thalamo-cortical circuit (cerebellar network), which is expected to contribute to IMSL in parallel to the cortico-basal ganglia-thalamo-cortical circuit (basal ganglia network) (Doyon et al., 2003; Hikosaka et al., 2002). However, our conclusion regarding network-wide modulation via cerebellar stimulation is supported by a recent simultaneous tDCS-fMRI study by Liebrand et al. (2020). Although they employed an explicit SRT task, the authors reported that tDCS to the right cerebellum, but not the left primary motor cortex, led to enhanced learning with increased learning-specific activity in the right primary motor cortex, cerebellum lobule VI, left inferior frontal gyrus and right inferior parietal lobule, compared to sham. Consequently, they concluded that cerebellar tDCS can influence functional activity and connectivity in the integrated motor learning network, comprising the cerebellar and basal ganglia networks. Future simultaneous tDCS-fMRI research should similarly investigate the impact of cerebellar tDCS on an implicit SRT task, to further elucidate the exact neural mechanisms underlying these complex effects.

## 5 Conclusion

Here, we investigated the impact of anodal cerebellar tDCS on the acquisition, short-term and long-term consolidation of IMSL, as measured by the SRT task. Our findings of increased sequence-specific learning effects, but not general learning effects, during anodal tDCS compared to sham, confirm the cerebellum’s involvement in the implicit acquisition of sequential knowledge. Additionally, the observed impact on accuracy during the presentation of unexpected stimuli (sequence deviants) corroborates the well-established role of the cerebellum in error-correction processes. These results provide compelling evidence that the cerebellar network not only plays a role in error correction processes, but also serves a sequence-specific function within the motor learning network. Given the consistency of this positive effect on IMSL across cerebellar (the present study) and primary motor cortex tDCS (our previous studies), we conclude that tDCS can beneficially modulate the integrated motor learning network that connects the basal ganglia and cerebellum at the cortical and subcortical level. Future simultaneous tDCS-fMRI studies are warranted to further elucidate the neural mechanisms underlying these effects.

## Data Availability

The raw data supporting the conclusions of this article will be made available by the authors, without undue reservation.
